# PreciseEdge raster RGB image segmentation algorithm reduces user input for livestock digital body measurements highly correlated to real-world measurements

**DOI:** 10.1371/journal.pone.0275821

**Published:** 2022-10-13

**Authors:** M. Jennifer Woodward-Greene, Jason M. Kinser, Tad S. Sonstegard, Johann Sölkner, Iosif I. Vaisman, Curtis P. Van Tassell

**Affiliations:** 1 USDA-ARS-NEA Animal Genomics and Improvement Laboratory, Beltsville, MD, United States of America; 2 Bioinformatics and Computational Biology Department, George Mason University, Manassas, VA, United States of America; 3 Department of Computational and Data Sciences, George Mason University, Fairfax, VA, United States of America; 4 Recombinetics at Acceligen, St. Paul, Minnesota, United States of America; 5 BOKU University of Natural Resources and Life Sciences, Vienna, Austria; European Commission, ITALY

## Abstract

Computer vision is a tool that could provide livestock producers with digital body measures and records that are important for animal health and production, namely body height and length, and chest girth. However, to build these tools, the scarcity of labeled training data sets with uniform images (pose, lighting) that also represent real-world livestock can be a challenge. Collecting images in a standard way, with manual image labeling is the gold standard to create such training data, but the time and cost can be prohibitive. We introduce the PreciseEdge image segmentation algorithm to address these issues by employing a standard image collection protocol with a semi-automated image labeling method, and a highly precise image segmentation for automated body measurement extraction directly from each image. These elements, from image collection to extraction are designed to work together to yield values highly correlated to real-world body measurements. PreciseEdge adds a brief preprocessing step inspired by chromakey to a modified GrabCut procedure to generate image masks for data extraction (body measurements) directly from the images. Three hundred RGB (red, green, blue) image samples were collected uniformly per the African Goat Improvement Network Image Collection Protocol (AGIN-ICP), which prescribes camera distance, poses, a blue backdrop, and a custom AGIN-ICP calibration sign. Images were taken in natural settings outdoors and in barns under high and low light, using a Ricoh digital camera producing JPG images (converted to PNG prior to processing). The rear and side AGIN-ICP poses were used for this study. PreciseEdge and GrabCut image segmentation methods were compared for differences in user input required to segment the images. The initial bounding box image output was captured for visual comparison. Automated digital body measurements extracted were compared to manual measures for each method. Both methods allow additional optional refinement (mouse strokes) to aid the segmentation algorithm. These optional mouse strokes were captured automatically and compared. Stroke count distributions for both methods were not normally distributed per Kolmogorov-Smirnov tests. Non-parametric Wilcoxon tests showed the distributions were different (p< 0.001) and the GrabCut stroke count was significantly higher (p = 5.115 e^-49^), with a mean of 577.08 (std 248.45) versus 221.57 (std 149.45) with PreciseEdge. Digital body measures were highly correlated to manual height, length, and girth measures, (0.931, 0.943, 0.893) for PreciseEdge and (0.936, 0. 944, 0.869) for GrabCut (Pearson correlation coefficient). PreciseEdge image segmentation allowed for masks yielding accurate digital body measurements highly correlated to manual, real-world measurements with over 38% less user input for an efficient, reliable, non-invasive alternative to livestock hand-held direct measuring tools.

## Introduction

### Manual body measures for trait prediction

Trait prediction from animal body measurements taken manually or from images is not a new concept [1–3]. The expense and inconvenience of using livestock scales to record weights [4–6] has inspired decades of research into alternative methods to obtain reasonably accurate weights for use in health, production and marketing livestock [4, 7, 8]. Body weights are important for many decisions in livestock health, production, and marketing, and body size is a preferred criteria to select breeding females, and conformation for breeding males in some developing regions [9]. Weight gain is dependent on age, stage of lactation or gestation, nutritional or disease status, and breed [10], and may inform traditional animal husbandry breeding and production decisions. In genomics and genomic tool development, physical measurements such as size and shape may be associated with productivity or with adaptive genes for traits such as milk production, fertility, disease and parasite resistance, or growth rate. Alternatively, physical measurements of size have long been used to estimate weight cheaply [4, 6, 7, 11–13]. Weight prediction formulas generally use some combination of chest girth, body length, or height at the withers to predict body weight (BW) [14, 15]. Conversion tables are available online for producers to predict body weights based on chest girth measures [14, 16, 17].

Researchers as early as the 1940’s and 1950’s investigated the most cost and time effective methods for gathering livestock body measurements for accurate body weight prediction. A critical research question was not only which body measures were the best predictors of actual body weight, but given that animals will naturally move between repeated measurements, causing the measurements to vary, how many times and how many observers should take the measurements to ensure accuracy? Touchberry and Lush in their 1950 paper "The accuracy of linear body measurements of dairy cattle", addressed the contradictory evidence at the time of whether the time and expense of taking multiple linear body measurements over time or observers was worth the effort. They gathered five common body measures (wither height, chest depth, body length, heart girth, and paunch girth) over 15 years, taking each measurement three times at specific ages. (Measurements as number of animals:age were 367:6 mo. 348:1 yr., 329:2 yr., 244:3 yr., 161:4 yr., 108:5 yr., and 38: 7 yr.) They found that assuming there was no egregious error in the manner of taking measurements, that while a small reduction in accuracy was possible, a single measurement is accurate for practical purposes [18]. The minimal variation between observers and thus the validity of using one observer to take livestock measurements has stood the test of time [19, 20]. In fact, even in the field of anthropometry (the study of human external morphological trait measurement) it has been shown that intra and inter-observer measurements are very similar, and thus the costs in time and effort for multiple observers adds little statistically to the results [21–23]. We describe collection of manual body measures on goats to use in comparison to digitally extracted body measurements from images. Due to the evidence outlined above, the manual measurements for this study were taken by one highly trained observer. This approach is expected to minimize variation while providing high accuracy in the manual body measurement values used to compare digitally extracted body measures.

### Digital image body measures for trait prediction

Image segmentation provides an essentially non-invasive data collection method on a broad range of scales [24]. It is often focused on collecting or analyzing morphology data, from remote sensing used to assess large land mass features, or in diagnostic medical imaging from a whole body, to organs, down to cells or for imaging plants to measure height, shape, size or color of plants or their flowers or grain heads. Measurements taken over time can provide dynamic phenotype information such as growth rates and more [25–27]. Image segmentation can be used to separate a feature or region of interest (e.g. subject animal) from the background using techniques involving intensity, color, edge, and texture [28]. The PreciseEdge algorithm is designed for image segmentation to enable extracting biologically relevant animal body measurements for developing predictive models of important livestock traits, such as body weight.

Non-invasive image measuring tools can be useful in a variety of situations where physical measurements are difficult because of distance, or that could be disruptive, dangerous, or damaging to the object or organism being measured. This is particularly relevant when working with live animals. Applications using digital images hold great promise for the future of high throughput phenotyping systems in livestock, but are in the early stage of development [29]. PreciseEdge image segmentation algorithm aims to reduce user input compared to GrabCut image segmentation algorithm, while maintaining or improving image edge detection precision. The highest edge detection precision is required so that digital measuring of the subject region of interest (ROI) will be an accurate representation of the subject in real life.

**Fig 1:**
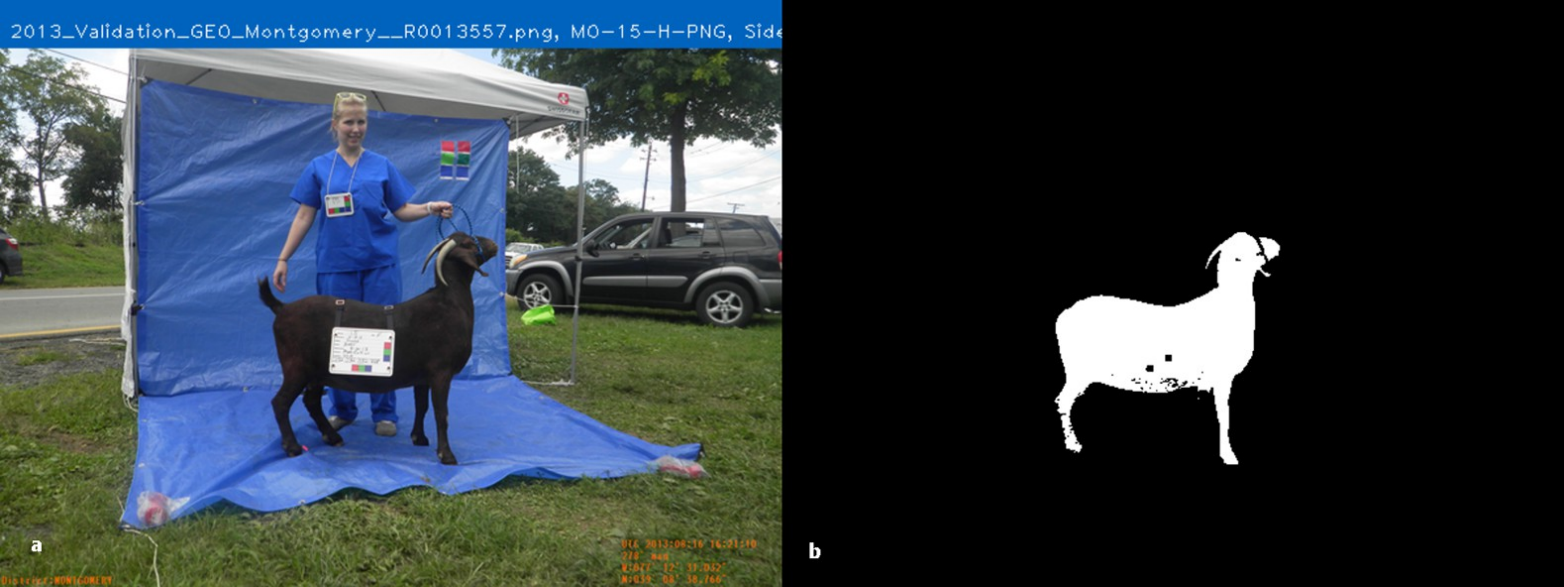
Image Matting, a) original image, b) alpha matte or mask image.

Image segmentation has many described approaches in the literature, often the modification or generation of basic techniques to address a unique image segmentation challenge, rather than a broad range of problems [25, 30]. The two major approaches to image segmentation include user defined backgrounds, and natural settings backgrounds [31]. Further, image segmentation can be either a fully automated process, or a procedure that requires some level of user input. The concept of capturing people or objects in the foreground of images with a user defined solid blue background to remove and replace the blue background with images from other scenes was applied in the movie industry in the 1930’s, long before the advent of digital images and video. The technique has been refined over the years, and applied to digital photography [32]. PreciseEdge is inspired by this concept, called the ’chromakey’ technique of ’matting’ an image (Fig 1). The purpose of chromakey is to make a composite of two or more video streams so well integrated that they appear to have been captured in a single scene in real life. In contrast, PreciseEdge removes a blue background without replacement, to clarify the ROI edges and enhance the results of the GrabCut algorithm by reducing user input (mouse strokes). The PreciseEdge algorithm segmentation mask allows measurements extracted from the image that correspond closely to real-life measurements of the animal in the photograph. This accurate correspondence of measures derived from digital images to the animal pictured is essential for digital images to be relevant in non-invasive, high-throughput digital phenotyping, and for development of measuring tools that can inform decisions on animal health and productivity. Such tools have the potential to minimize animal handling required to gather measurements, which in turn may reduce animal stress or discomfort. These changes could then result in more accurate and consistent measurements compared to manually collected data.

### State-of-the-art in automated body measures

Machine learning methods to train models to extract livestock body measurements automatically from digital image data are beset with three major challenges around acquiring labeled image training data sets, namely 1) the labeled data sets are not readily available in large enough numbers, 2) the labeled (mask) data sets are not of the precision required for extracting body measurements similar to real-world body measurements, and 3) high quality labeled image data sets can be generated manually, but this is time consuming and expensive [26, 33–36]. The technology and automation is advancing, however high precision for livestock body measurements still requires some manual inputs [37–39].

Segmentation for simple classification is trivial in comparison to segmentation intended to directly extract complex measurements for further analysis to predict traits with real-world impact. One example is in particle image velocimetry (PIV) for multiphase flows where such imprecise segmentation could have environmental implications in enhanced oil recovery or ground water remediation work and more [40]. Similarly in livestock, precision is required, and the interest in this paper is to extract accurate body measures of live animals from images.

Refinement of current methods for measuring or examining animals in a non-invasive way, could improve animal welfare and productivity, the safety of both animals and humans, as well as improving the measurements themselves used to make animal health and welfare decisions. Animal measurements taken by hand are notoriously variable across different observers and this is mitigated by using only one observer with negligible statistical penalty. As described in the section above ’Manual Body Measures for Trait Prediction’, and specifically examined by Touchberry and Lush, this variation is due to animal movement between repeated measures more so than human error [18]. Digital tools will provide consistency across sampling situations. Therefore, in the case of measurements taken by many individuals across different locations, such tools are sorely needed. A particular challenge is photographing animals in a wide array of locations, including fields, barns, or even in wild or natural settings. Establishing a reasonable correlation between measurements extracted from images and real-world biological significance is a logical first step to ensure development and adoption of digital tools that will deliver data and analysis for those studying or caring for animals.

Manually generated image annotations are the gold standard for developing machine learning training data. The manual methods often start out with a bounding box drawn by a user with the computer mouse, to identify the object of interest and simultaneously, to identify the background pixels outside of the bounding box. Some workers have attempted to automate drawing the bounding box [41] and for livestock, combining that with convoluted neural networks (CNN) and standardized cow images found online [42, 43]. This method provides promising segmentation; however, the values are ’relative’ but not directly related to real-world biological measures. Additionally, the cattle images used were found online and show the animals in a relatively consistent pose, they do not represent real-world conditions. These are professional marketing photos that are often retouched or include fake backgrounds which likely impact image segmentation. Still other researchers used machine generated bounding boxes from video to train CNN models with only the best masks moving from one model generation to the next [34]. Developing CNN models to target select features have also been applied [44]. However, these attempts still overlook the problem of needing a large supply of consistently taken images that also represent a variety of animals in the real-world to develop automated tools to extract digital body measurements.

### GrabCut algorithm

The GrabCut algorithm begins with a user drawing a bounding box around the region of interest (ROI), and it was developed to segment images with highly complex background features, as in a natural environment using graph-based segmentation[45]. A modified version of GrabCut is integrated into the PreciseEdge algorithm, whereby only the ’sure foreground’ and ’sure background’ data is identified and input into the GrabCut algorithm leaving out the ’possible foreground’ and ’possible background’ data described in the original GrabCut paper. The base techniques employed by GrabCut are graph cuts and border matting. Matting is the generation of a binary mask image or ’alpha matte’ that shows the foreground opacity, and results in an image where the foreground ROI pixels are shown in white, and the background pixels are shown in black [31]. Mask pixel values are 1 for white and 0 for black, and the mask can be multiplied with the original image to leave only the foreground, or ROI (Fig 1). This process is done by segmentation of an ROI, or foreground object by estimating the background color, and the foreground color and opacity (alpha matte) interactively, with the user marking the foreground and background manually on the image with the computer mouse [46]. GrabCut begins with iterative graph cuts, and then refines that segmentation with border matting around the likely edges identified by graph cuts, reducing the amount of user interaction required, and regularizing the alpha values in its border matting step, which reduces visible artifacts [47].

### PreciseEdge algorithm

PreciseEdge addresses the problems of quantity and quality of labeled images, including reducing the user input required to develop them, from image collection to image processing and data extraction. We add a pre-processing step to a GrabCut inspired by ChromaKey to reduce user input required by GrabCut. A fully automated image processing algorithm to extract body measurements directly from the images was used on output masks from GrabCut-only and PreciseEdge for comparison. PreciseEdge combines human and automated inputs to generate labeled images that are so precise, that digital measures can be taken directly from the image rather than via model generated predictions. We describe in detail how PreciseEdge uses a two-step image segmentation to prepare image masks for automated extraction of digital measurements for accurate body measures. To apply PreciseEdge, all images are collected with the African Goat Improvement Network Image Collection Protocol (AGIN-ICP) we developed [48, 49].

## Materials and methods

Individuals holding goats depicted in the images included in this manuscript, supplementary materials, and data have given written informed consent (as outlined in PLOS consent form) to publish these images.

### Image capture and data extraction

Seventy-five goats were carefully measured by hand with a tape measure by one highly trained technician and photographed at public animal shows with written permission of the owners. The photo set up and posing of animals were done according to the African Goat Improvement Network Image Collection Protocol (AGIN-ICP) [49], which includes a sign for size and color calibration to be included in the images and blue plastic tarps behind and beneath the subject goat. A Ricoh Digital camera was used, and high and low light levels for four sets of two poses for each sample animal for a total of 300 images. The rear and sign poses were used to generate masks with both the PreciseEdge or GrabCut image segmentation algorithm to create image masks for extracting body measures and calibration data. Image region of interest (ROI) measures extracted were converted from pixel count values to centimeters using the extracted calibration sign measurements. Calibration signs are rectangular, and thus easily extracted using GrabCut, and the measurements were converted to centimeters to convert extracted digital body pixel measurements.

### Compression format—JPG conversion to PNG

It is not always mentioned, but images processed through any analysis are impacted by the image compression method, which can cause a loss of data making the file smaller and easier to store, but that may lose precision in image segmentation. The predominant image format used by digital cameras today is JPG, also known as JPEG. These files are RGB raster files using lossy compression to produce a smaller file needing less storage space and that is easier to share across the internet. Lossy compression uses the discrete cosine transform (DCT) [50], where high frequency data are removed, so are lost in the compression. The resulting image is typically acceptable to the human eye, as people cannot perceive the missing data. However, to obtain accurate measurements from images, this type of unrecoverable data loss is problematic [51]. Therefore, a lossless compression for the raster RGB image, such as the portable network graphics (PNG) [52] would preserve the image data. PNG compression is the chief lossless format found on the internet. For PNG compression, the image is first passed through a filter, where each pixel is represented with its value difference from an adjacent pixel for each color channel (red, green, blue), for each row of pixels in the image. These are then compressed, row-by-row based on pattern matching of these differences. Each pattern is saved on the first instance, and then that instance is referred to whenever it is repeated in the image [53]. The type of filtering and the number of patterns within each image will vary the compression ratio, or level of reduction in size.

Testing image compression formats of JPG (as the images come out of the camera) versus conversion to PNG format was conducted. JPG images were converted to PNG images by changing the image file name extensions from JPG to PNG. Automated capture of user input was done by counting pixel points (x, y) locations marked by the user on each image in GrabCut-only and PreciseEdge Step 2, and each point counted as one ’stroke’ of the mouse. Mask outputs, and mean stroke counts were compared for JPG versus PNG images, and it was determined that PNG images result in increased mask precision, and reduced user input for both segmentation algorithms. Therefore, for the following comparison of GrabCut versus PreciseEdge algorithms, all images were processed using PNG format.

### ROI isolation with GrabCut and PreciseEdge

**Fig 2:**
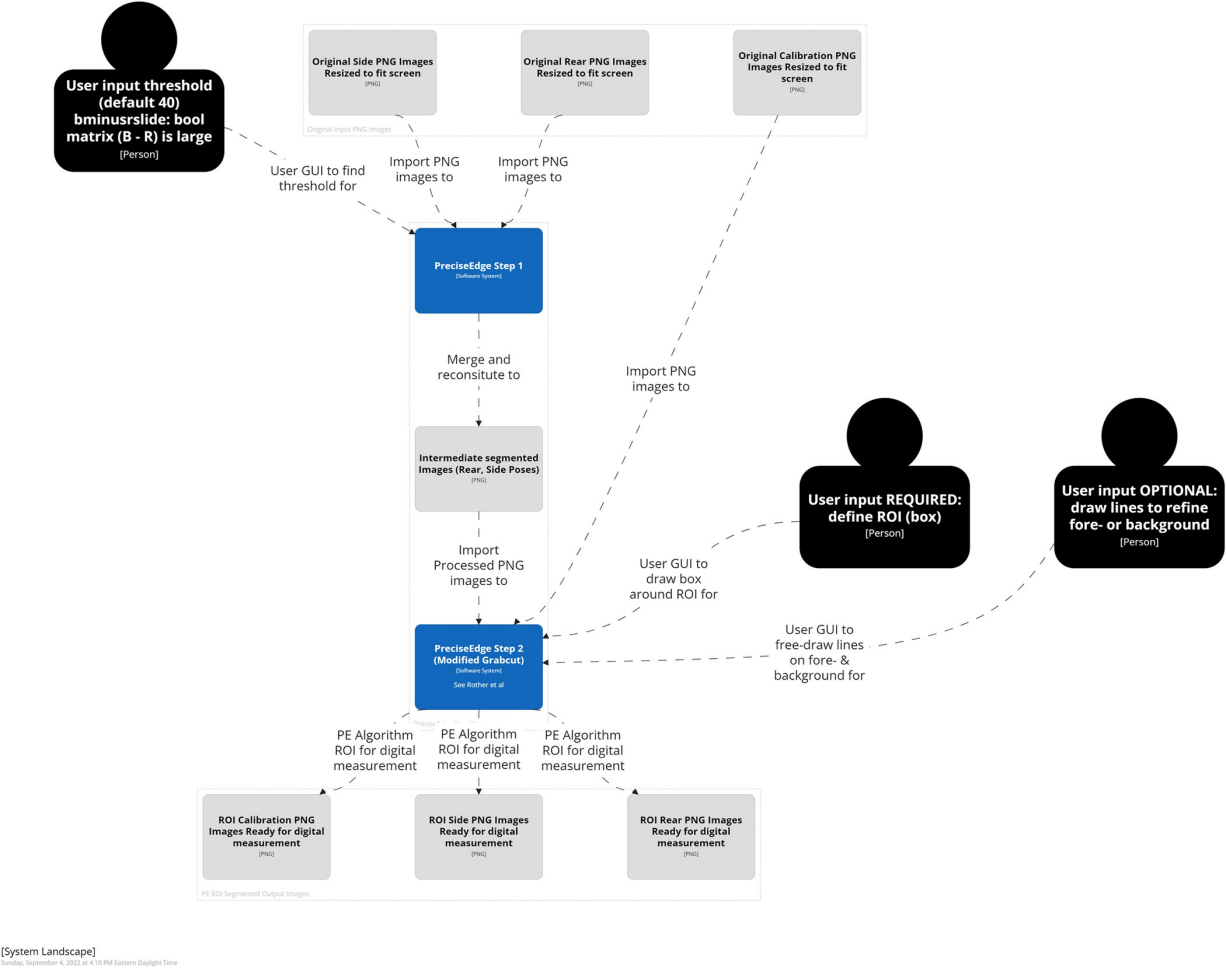
PreciseEdge algorithm overview.

**Fig 3:**
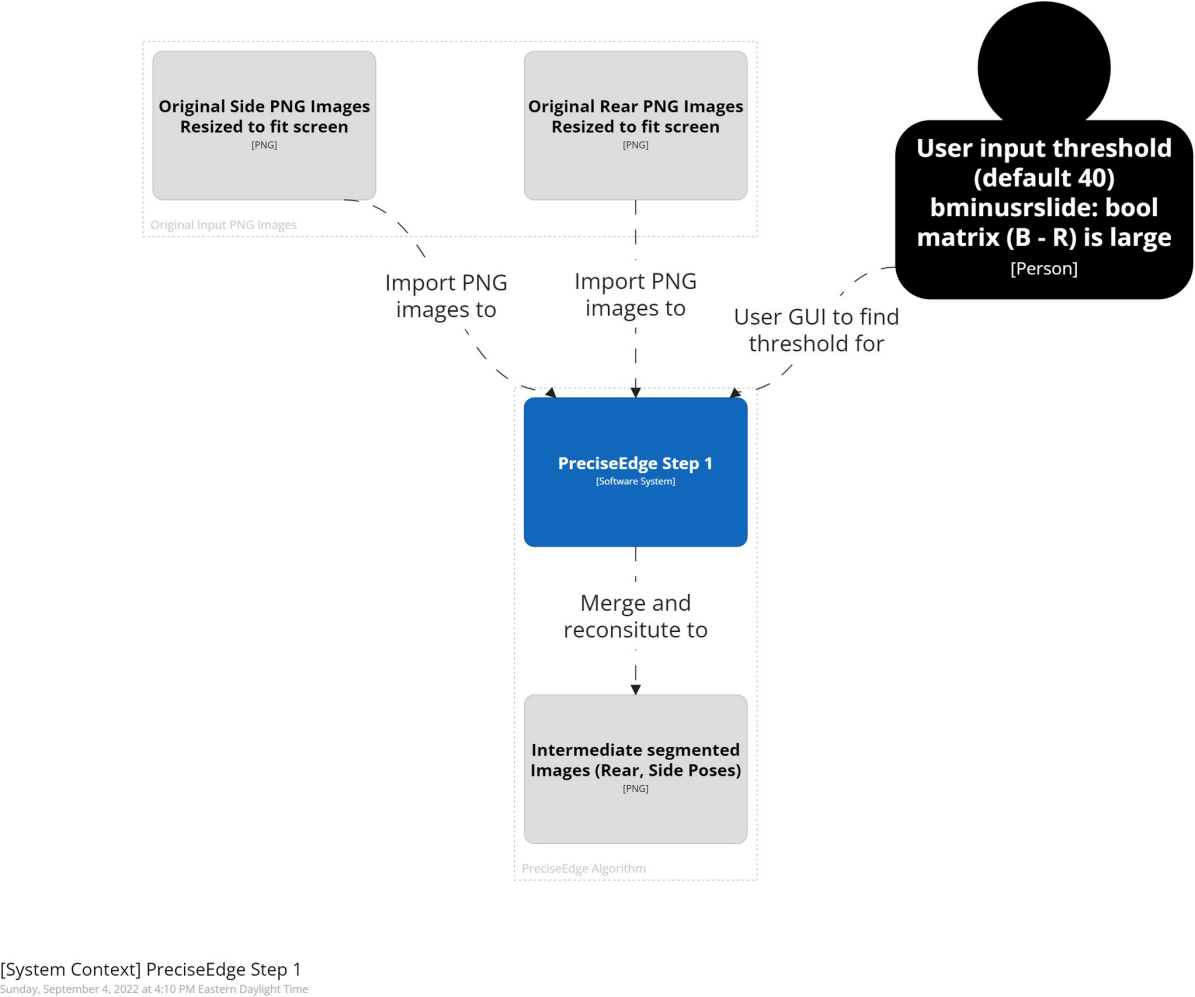
PreciseEdge algorithm step 1 overview.

**Fig 4:**
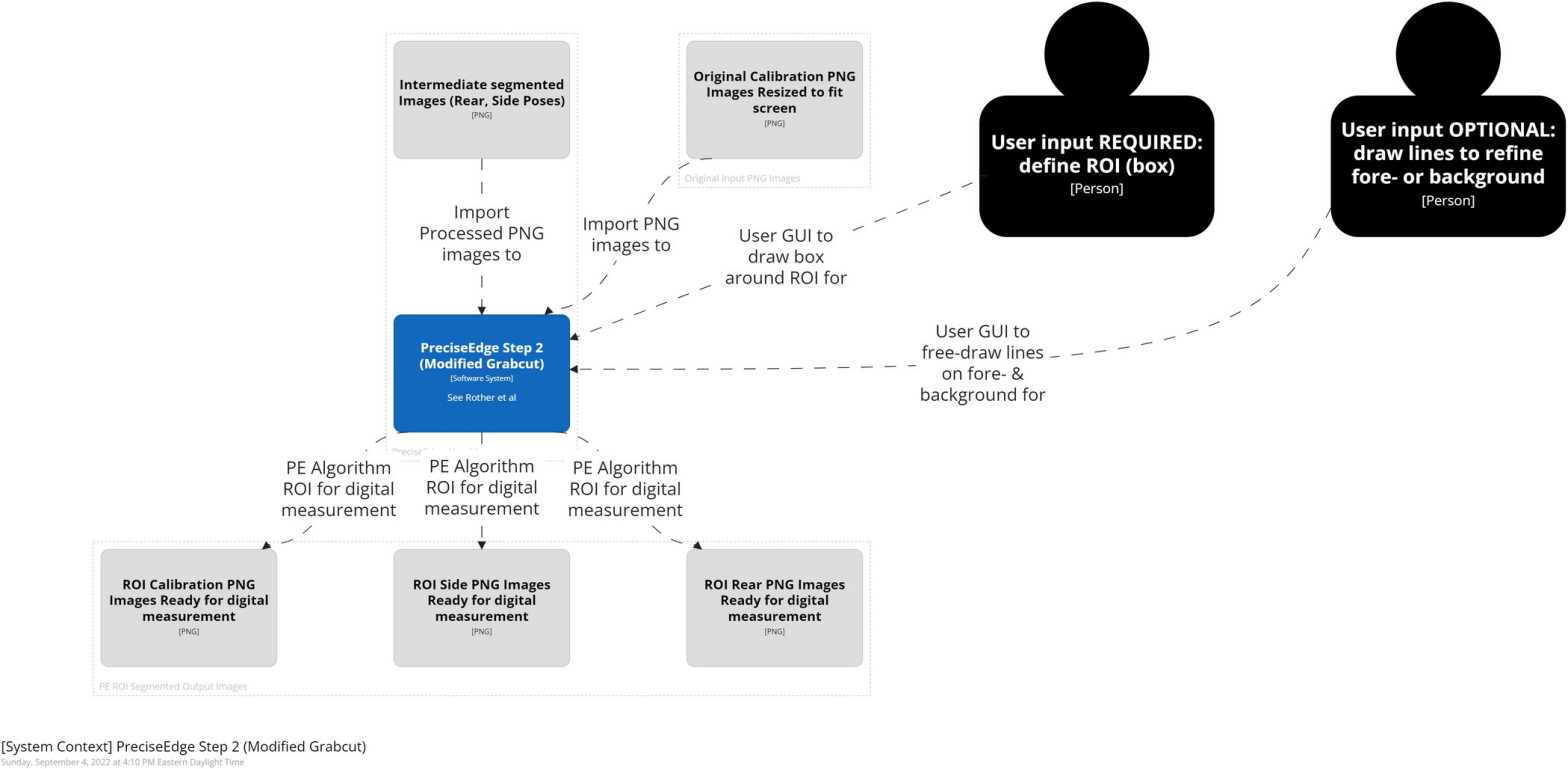
PreciseEdge algorithm step 2 overview.

**Fig 5:**
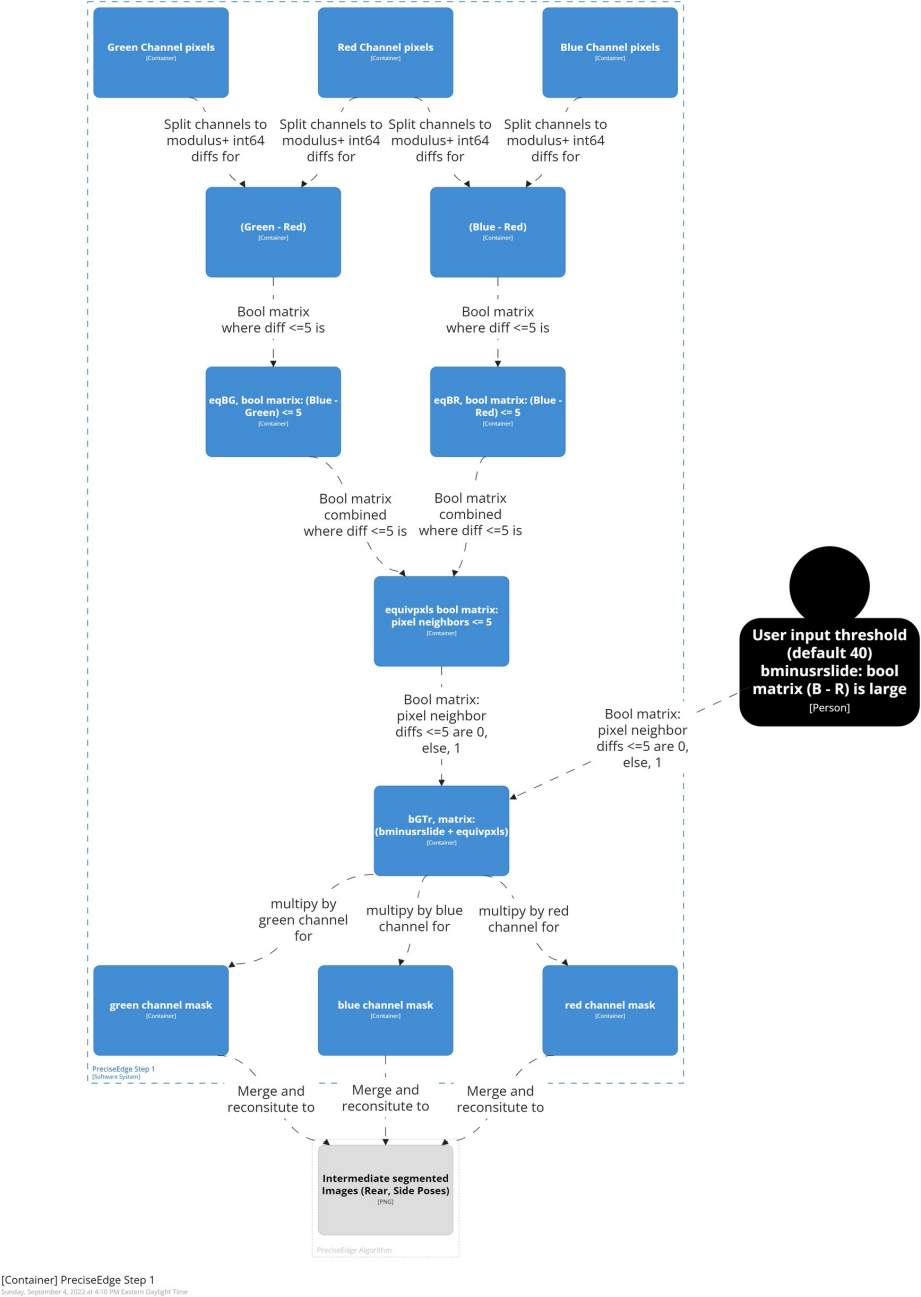
PreciseEdge algorithm step 1 detail.

**Fig 6:**
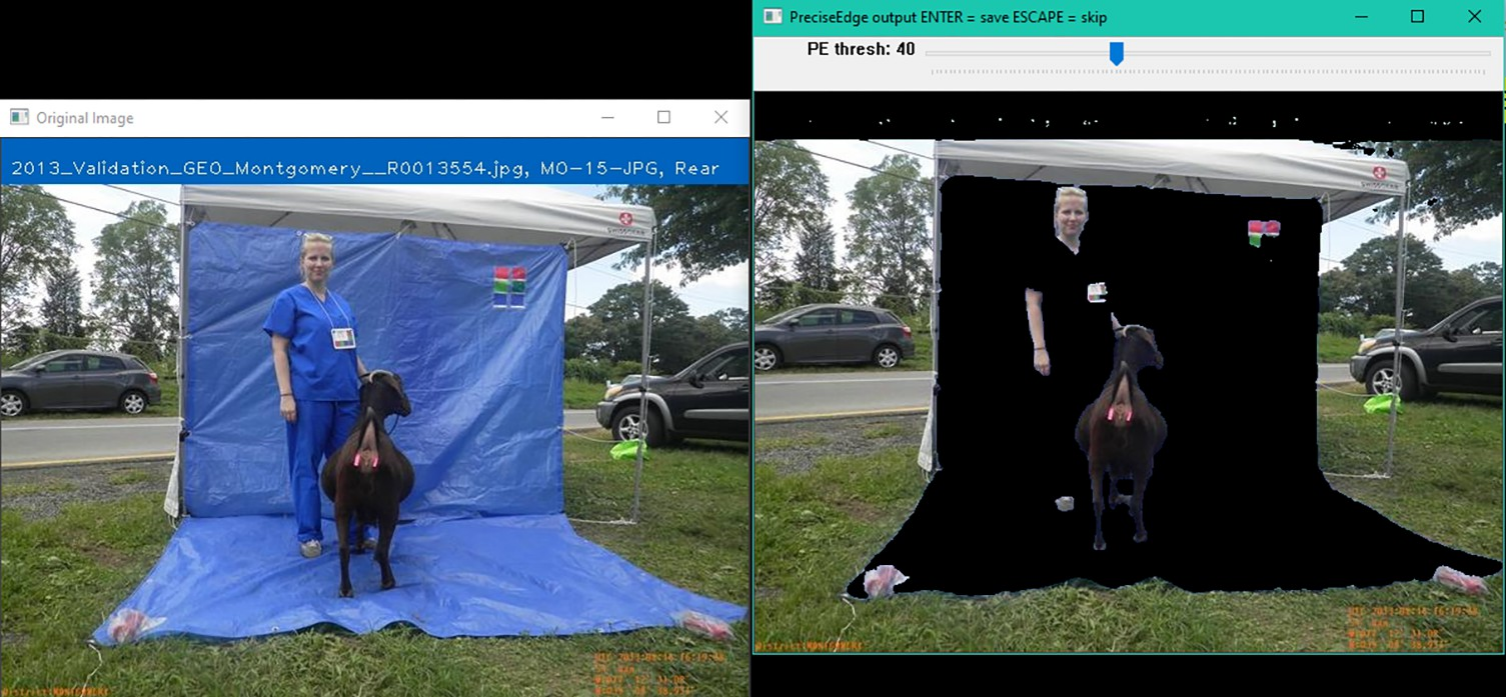
PreciseEdge Color Step GUI, reference image (left), interactive image (right).

The PreciseEdge algorithm (Figs 2–5) is a two-step process for user input. Step 1 is a color step removing the blue background in the image (Fig 6). Step 2 uses the output image from Step 1 and applies GrabCut to further refine the ROI. Once the ROI is identified, the images are subjected to fully automated processes to complete the refinement of the mask, image labeling, and measuring the animal’s body and generation of output images and data files. Further refinement was done using OpenCV morphologyEx functions [54].

To compare PreciseEdge and GrabCut, only the PreciseEdge images went through the color step, (Step 1, Fig 6), as a pre-processing step inspired by chromakey. Chromakey separates the image foreground and background so the background can be removed and replaced with another scene from a different image for dramatic effects. In contrast, PreciseEdge works with just one image with the goal of producing an output image that ’helps’ the GrabCut process separate the foreground and background. GrabCut-only images skipped the color step, Step 1, and used the original full color images for Step 2 (S1 Table).

Three hundred images were processed through the same software with the only difference for PreciseEdge being that the images went through the brief, pre-processing chromakey color step (Figs 3 and 5). Post-ROI processing for generating masks and measures was identical, and fully automated for both algorithms. The images were taken with a Ricoh digital camera (JPG), outside or inside barns, of 75 goats pictured from the side and rear in two different settings each, for four images per goat. The collection procedure images included a custom calibration feature to transform pixel measurements to real world measures. The goats were manually measured, weighed and photographed per the AGIN-ICP. To mitigate variation, all the manual measures were taken by one highly trained person, and the digital measures user input were also taken by one highly trained person. Digital measures were correlated with the three most common manual livestock body measures of body height, length, and chest girth.

Images from digital cameras (usually JPG format) input to PreciseEdge are first resized so they will fit on the computer screen and their extensions changed to PNG for processing. The first step in the PreciseEdge algorithm is inspired by chromakey (Figs 3 and 5), and the second step is a modified GrabCut (Fig 4), with user input identifying the ’sure’ foreground and background data, and not using ’probable’ data as outlined in the original GrabCut paper.

#### PreciseEdge—step 1

In step one (Fig 5), the resized color image (RGB) is split into its individual color channels. Image pixels are arranged in three spatial dimensions, *(k = 3)* with the vertical and horizontal planes represented as *i* and *j*, respectively. Each *k* dimension represents intensity values for red *(R)*, green *(G)*, and blue *(B)* color channels, respectively (Eqs 1, 2, and 3).


$$r_{ij}={RGB}_{ij}\forall_{ij}$$ (1)

$$g_{ij}={RGB}_{ij}\forall_{ij}$$ (2)

$$b_{ij}={RGB}_{ij}\forall_{ij}$$ (3)

Each channel array is changed from the OpenCV default image number type, unsigned integer ‘uint8’ *(0–255)*, to integer ‘int64’ *(-2*^*63*^ to *2*^*63*^) for signed integer calculations (Eqs 4, 5, and 6), where *R*, *G*, and *B* are the color channels, red, green, and blue, and *r*_*ij*_, *g*_*ij*_, and *b*_*ij*_ are individual pixel intensity values in each channel. The resulting values from converting unsigned values will be positive, thus the modulus is the positive range for ‘int64’ (*0* to *2*^*63*^ scale).


$$R_{ij}=[r_{ij}\;mod(2^{63}+1)]\forall_{ij}$$ (4)

$$G_{ij}=[g_{ij}\;mod(2^{63}+1)]\forall_{ij}$$ (5)

$$B_{ij}=[b_{ij}\;mod(2^{63}+1)]\forall_{ij}$$ (6)

Matrices of difference values between channels, (Eqs 7 and 8), where *R*, *G*, *B* are the ‘int64’ transformed channels, are created,

$${BR}_{ij}=[B_{ij}-R_{ij}]\forall_{ij}$$ (7)

$${GR}_{ij}=[G_{ij}-R_{ij}]\forall_{ij}.$$ (8)

Pixels are identified, where *B* and *R*, and *G* and *R* are all close in value which will retain red hues—browns, oranges, etc. Boolean matrices are created for *B* to *R (eqBR*_*ij*_*)*, and *B* to *G (eqBG*_*ij*_*)* equivalencies whereby a value of *1* is given for pixel locations with small difference values of *5* or less, and the value of *0* is given otherwise (Eqs 9 and 10).


$${eqBR}_{ij}=\left\{ \begin{array}{c} 1(x_{i},y_{j})\in Relation(\leq 5)\\ 0(x_{i},y_{j})\notin Relation(\leq 5) \end{array}\right.\forall_{ij}$$ (9)

$${eqBG}_{ij}=\left\{ \begin{array}{c} 1(x_{i},y_{j})\in Relation(\leq 5)\\ 0(x_{i},y_{j})\notin Relation(\leq 5) \end{array}\right.\forall_{ij}$$ (10)

The truth value is computed of the Boolean equivalencies to a matrix “*equivpxls*” to identify pixel locations with a value of *1*, where all three *(R*, *G*, *B)* values are within *5* of each other, and represented by a zero otherwise (Eq 11).


$${equivpxls}_{ij}=\left\{ \begin{array}{c} 1({eqBR}_{ij},{eqGR}_{ij})\in Relation(1,1)\\ 0({eqBR}_{ij},{eqGR}_{ij})\notin Relation(1,1) \end{array}\right.\forall_{ij}$$ (11)

The locations where blue difference from red *(B–R)* is large are identified, and a graphical user interface (GUI) is set up so the user can specify and test a *(B–R)* range lower threshold for each image, and then save the finished image. The user is shown the output name for the finished image, and the image is displayed in a window, “PreciseEdge output”, for real-time processing by the user, see Fig 6.

A track bar is created to set the threshold on the window, 40 is the initial low setting, which works for most images, and thus requires only one-click confirmation by the user. The highest possible value is 120, the lowest is zero. A variable is created to capture the final track bar position when the user saves the image. A Boolean matrix, called “*bminusrslide*” is created, where the *(b-r)* difference values are large, (between the track bar position and *256*) are set to zero, all others are set to one (Eq 12).


$${bminusrslide}_{ij}=\left\{ \begin{array}{c} 1({bminusr}_{ij})\notin Relation(track\;bar\;value\leq 256)\\ 0({bminusr}_{ij})\in Relation(track\;bar\;value\leq 256) \end{array}\right.\forall_{ij}$$ (12)

The matrices, *bminusrslide* and *equivpxls*, are added and the result called *bGTr* (*b* is greater than *r*) (Eq 13). This yields a Boolean matrix, *bGTr*, with values of zero where *b-r* is large, and a value of one where differences between *B*, *G*, and *R*, are all less than *5*.


$${bGTr}_{ij}=[{bnimusrslide}_{ij}+{equivpxls}_{ij}]\forall_{ij}$$ (13)

A color mask array is created for each channel, by multiplying each of *R*, *G*, and *B*, by the matrix *bGTr* (Eqs 14, 15, and 16). This will return values of zero for *x*_*i*_ *y*_*j*_ values in the channel positions where *b—r* is large (will yield black where the original image showed blue). It will retain the original intensity values where *b*, *g*, and *r*, are all within *5* intensity values of each other. Each mask is converted back to the original data type, unsigned integer, uint8 (*0–255* scale), for further processing.


$${rmask}_{ij}=\left[[R*{bGTr}_{ij}]mod\;256\right]\forall_{ij}$$ (14)

$${gmask}_{ij}=[[G_{ij}*{bGTr}_{ij}]mod\;256]\forall_{ij}$$ (15)

$${bmask}_{ij}=\left[[B_{ij}*{bGTr}_{ij}]mod\;256\right]\forall_{ij}$$ (16)

The image matrix is reconstituted by iteratively merging the mask matrices for each channel, (*k = 1*, *2*, *3)* i.e.[*rmask*], [*gmask*], [*bmask*], (Eq 17), and the user is shown the reconstituted image such that they can see it change as they select different values on the track bar.


$${bmask}_{ij}=\left[[B_{ij}*{bGTr}_{ij}]mod\;256\right]\forall_{ij}$$ (17)

#### PreciseEdge—step 2 (GrabCut)

**Fig 7:**
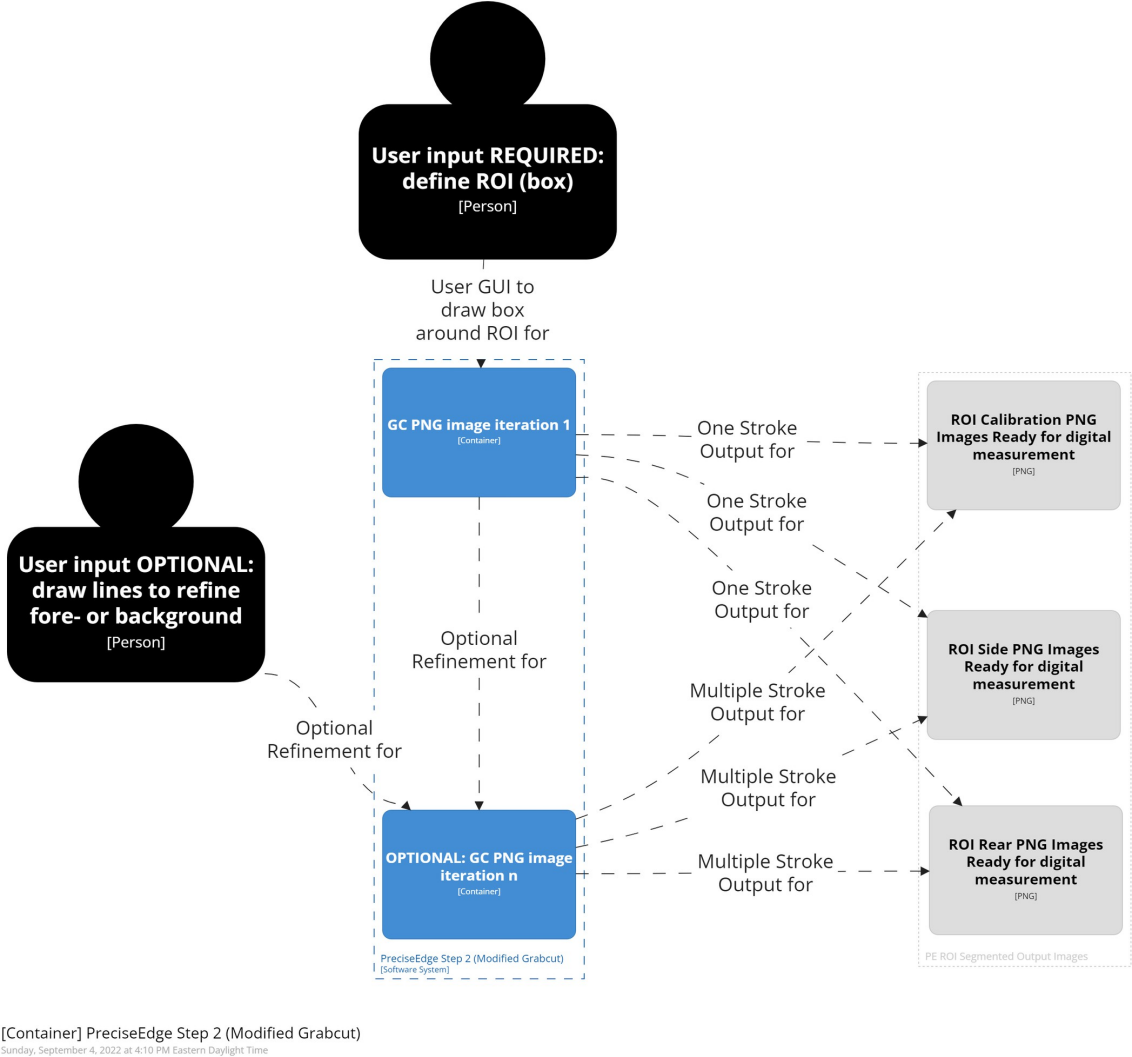
PreciseEdge algorithm step 2 detail.

PreciseEdge Step 2 employs GrabCut (Fig 7). For the GrabCut-only processed images, step 2 is the first step, and the original resized, and transformed to PNG images are input directly. For the PreciseEdge images, this is the second step, and the output images from step 1 (color step) are input to this GrabCut step. The color step generally takes seconds to perform as it is a quick view of the image by the user to ensure the default setting is adequate. It can easily be automated if images in the data set are uniform. We have implemented a modified version of the ‘GrabCut interactive foreground extraction using iterated graph cuts’ developed by Rother, et. al. [47]. The python code from the ‘grabcut.py’ sample on GitHub [55] was modified to omit the ’possible foreground’ and ‘possible back-ground’ matrices that are not needed for either method for this study. It also included minor changes to adjust user input i.e., mouse click/line coloring, and modifications to manage input and output of the individual and stacked images and filenames, based on the feature being isolated. Examples of input images for PreciseEdge step 2 are shown in the first image of each trio of images in the S1 Table in the center column ("Intermediate Images"). GrabCut-only testing also uses the identical modified GrabCut version, but the input images are the original images, note the blue background is intact, while the PreciseEdge input images show the blue background removed in the color step (Fig 6).

As each output image from PreciseEdge step 1 is processed with the modified GrabCut, the input image (output image from step 1), and the interactive output image is shown to the user for reference. The user draws a bounding box around the feature of interest. Once the box is set, the first iteration of GrabCut is run on the pre-processed image. If there are still some foreground or background incorrectly segmented in the first iteration, the 0 and 1 keys in combination with the mouse are used to identify foreground and background. The best results if this is needed, are obtained by drawing just inside the ROI along the edge for the foreground, and just outside of the ROI for the background. To compare user input for PreciseEdge and GrabCut, rear and side poses were run through GrabCut and PreciseEdge respectively.

### PreciseEdge and GrabCut comparison

To compare user input needed for PreciseEdge and GrabCut-only methods, the software was modified to capture the initial bounding box image for visual review (S1 Table "Intermediate Images"). This provides an indication of how much additional refinement users may need to do. To evaluate the intensity of refinement required under each method, the code was further modified to capture the pixel locations of each line drawn by the user with the mouse, to identify sure foreground and sure background before saving the image (S1 Table "Intermediate Images"). The pixel (x,y) coordinates added to each image were counted. Each pixel counted as one stroke, with a higher stroke count meaning that the user had to draw more or longer lines on the image to satisfactorily isolate the region of interest on each image. This is an indication of how much additional refinement was required for each image in the second step (GrabCut).

## Results and discussion

**Fig 8:**
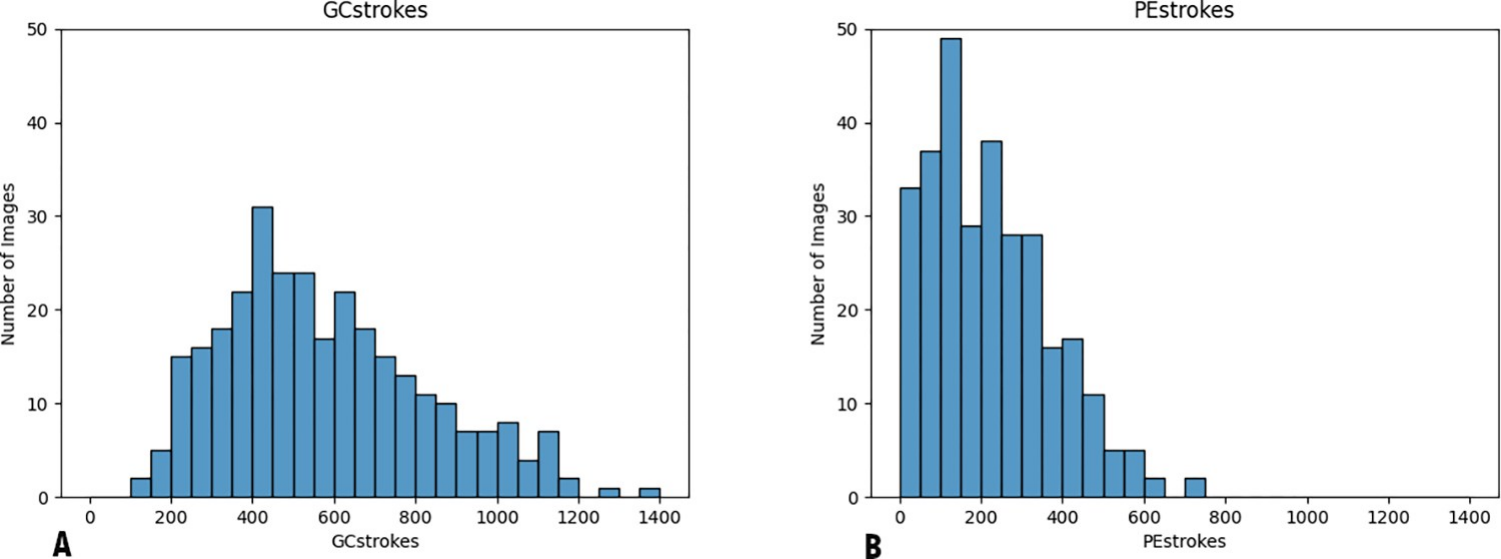
Histograms of GrabCut (A) and PreciseEdge (B) stroke count distributions, bin width is 50 (strokes). The distribution of stroke count for PreciseEdge shows more consistency of images needing fewer strokes (38% fewer) compared to GrabCut processed images.

The stroke counts were evaluated for normal distribution (Fig 8), and to determine if they were the same distribution by Kolmogorov–Smirnov tests. Non-parametric Wilcoxon tests were performed to show the difference between the GrabCut and PreciseEdge stroke counts.

To see if digital images ROI isolated by PreciseEdge and GrabCut would yield measures correlated to manually taken measures of the subject animals in real life, the digital measures output from each method were correlated to the manual measurements. Again, the post GrabCut masking and measuring processing was automated, and identical for each algorithm. Pearson correlations were done to compare the digital measures for body length and height, and for chest girth with the same measurements taken manually for the goat pictured.

We tested and confirmed that image format or compression type is also a factor in the precision of masks output whether using PreciseEdge or GrabCut. This is due to differences in lossy image compression in JPG developed by the Joint Photographic Experts Group (JPEG) [50], and lossless portable network graphics (PNG) [52] format. The lossy compression of JPG images results in data removed from the original image to reduce file size. PNG compressed images retain their information (lossless). A comparison of PreciseEdge with a sample in JPG and PNG formats is shown in images in S2 Table. The JPG and PNG to isolate the ROI showed PNG consistently returned a cleaner more precise mask (S2 Table).

**Fig 9:**
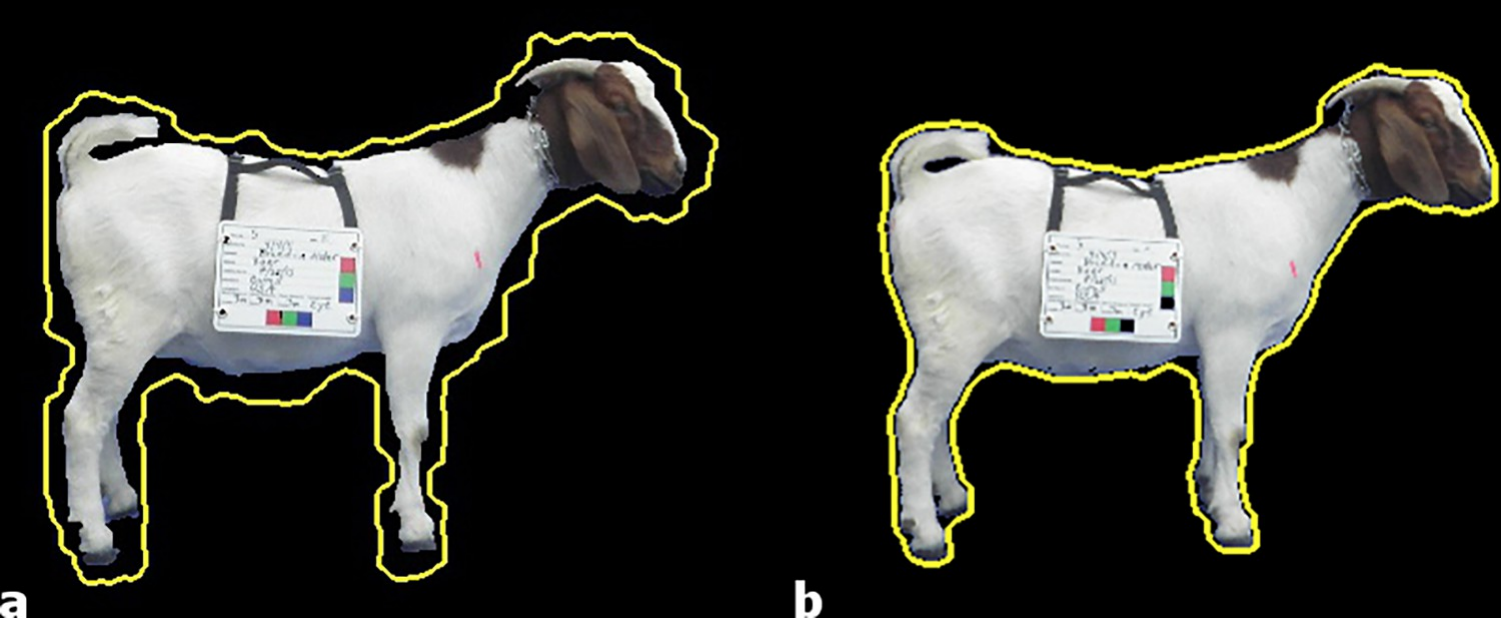
GrabCut JPG image (invisible artifacts around edges) (a) and PreciseEdge PNG image (b), edges detected by each approach (yellow line).

**Fig 10:**
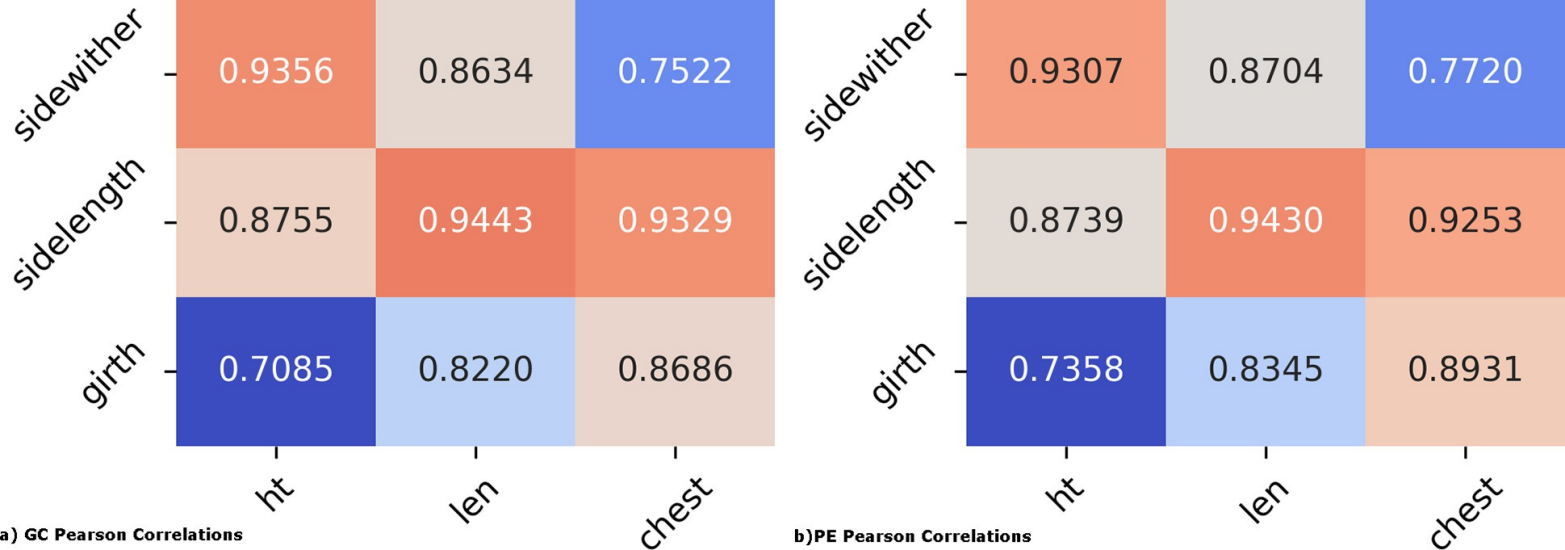
a) GrabCut (left) and b) PreciseEdge (right) correlations to body height and length and chest girth. Manual measures (ht, len, chest), digital measures (sidewither, sidelength, girth).

According to its authors, the GrabCut algorithm enhances prior art by removing “visible artifacts”, which is adequate for many applications [47]. However, they acknowledge in their paper that invisible artifacts may remain to produce “blocky” masking and edge detection, with true edges approximated. In working with GrabCut we discovered the artifact issues were only seen with JPG compression (Fig 9). The GrabCut paper [47] does not specify the type of image format to be used. We found that GrabCut ROI precision is significantly improved with PNG images over JPG images, and the ’blocky’ mask resolved. Thus, all images are converted to PNG prior to step 1 of PreciseEdge and for testing with GrabCut-only. The PreciseEdge segmentation produces a nearly identical segmentation results compared to GrabCut when inputting PNG images, but with significantly less (over 38%) user input. Pearson correlation coefficients for digital measures to manual measures of body height, length, and chest girth were high at (0.936, 0. 944, 0.869) for GrabCut and (0.931, 0.943, 0.893) for PreciseEdge (Fig 10).

With PreciseEdge, images require less user input beyond the initial drawing of the bounding box. With GrabCut, there is often some background under the belly or under the neck of the goat that needs to be removed by the user. The PreciseEdge step 1, color segmentation method requires minimal user input in the color segmentation step, and it is possible for it to be fully automated if the original input images are uniform and do not require varying segmentation thresholds. The GrabCut or PreciseEdge user input depends on the quality of the images, but the prior PreciseEdge color segmentation speeds up the GrabCut step considerably. The mean stroke count for GrabCut is 577.08, with a standard deviation of 248.45. PreciseEdge is significantly less with a mean stroke count of 221.57, and a standard deviation of 149.45 (p = 5.115 ^e-49^).

The output from the single iteration of GrabCut or PreciseEdge (bounding-box-only) demonstrates the difference in user input that would be needed to correct the mask and are shown in S1 Table. Table of Figures, input, process, and output comparison for GrabCut versus PreciseEdge ("Intermediate Images"). The "Output Images" show the final labeled images from GrabCut and PreciseEdge. The rear poses are the most challenging for edge detection due to the closeness of the rear legs to each other, and their individual narrowness. Comparing the bounding-box-only examples for both algorithms, demonstrates the additional user input needed for GrabCut-only versus PreciseEdge with the pre-processing color step. The trio images showing the bounding box plus additional user input required further illustrates the additional user input required to define the ROI using GrabCut-only versus PreciseEdge. Final digital body measurements were generated using the output ROI image from each algorithm as input for the identical, fully automated process to further refine, then measure and label each image for both GrabCut-only, and PreciseEdge. The final images shown in the column, "Output Images", illustrate the final output is similar for both algorithms.

## Conclusions

The PreciseEdge image segmentation algorithm was shown to produce masks that are precise enough to extract consistent, high quality animal body measurements directly from digital images. This was done with significantly less user input required (38%) than with GrabCut, and both PreciseEdge and GrabCut performed better using PNG images. PreciseEdge generated image masks used to extract measurements from digital images were highly accurate when compared with standard manual body measurements, and will allow important animal health and productivity data to be collected without bothering the animals. The increased speed of the image segmentation due to reduced user input and high correlation to actual animal body measurements made possible by the PreciseEdge image segmentation algorithm make this a realistic option for livestock. This can reduce animal and human stress while increasing the accuracy and consistency of measurements. Further, due to significantly reduced user input while maintaining the high quality of the labeled output images, we believe PreciseEdge image segmentation would be excellent for use in developing machine learning model training data to advance the field of automated livestock body measurement.

## Supporting information

S1 Table Table of figures, input, process, and output comparison for GrabCut versus PreciseEdge. (DOCX)

S2 Table Table of figures, PreciseEdge comparison of JPG and PNG input image formats. (DOCX)
