# Supplementary material for: PreciseEdge raster RGB image segmentation algorithm reduces user input for livestock digital body measurements highly correlated to real-world measurements
Source: PLoS One. 2022 Oct 13;17(10):e0275821. doi: 10.1371/journal.pone.0275821 (PMC9560539; doi:10.1371/journal.pone.0275821)

## **Supporting Information**

**Table 2. Table of Figures, PreciseEdge comparison of JPG and PNG input image formats.**


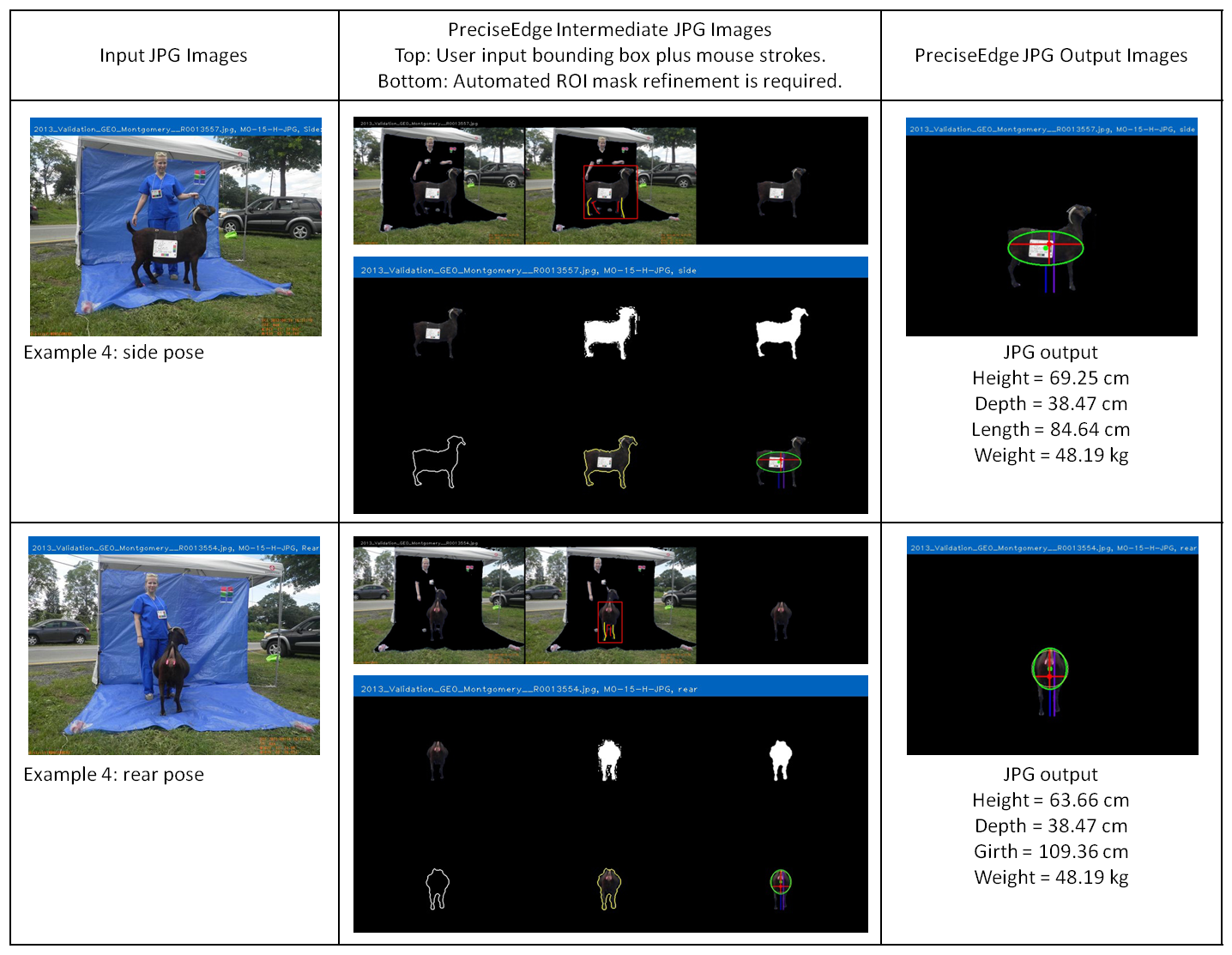

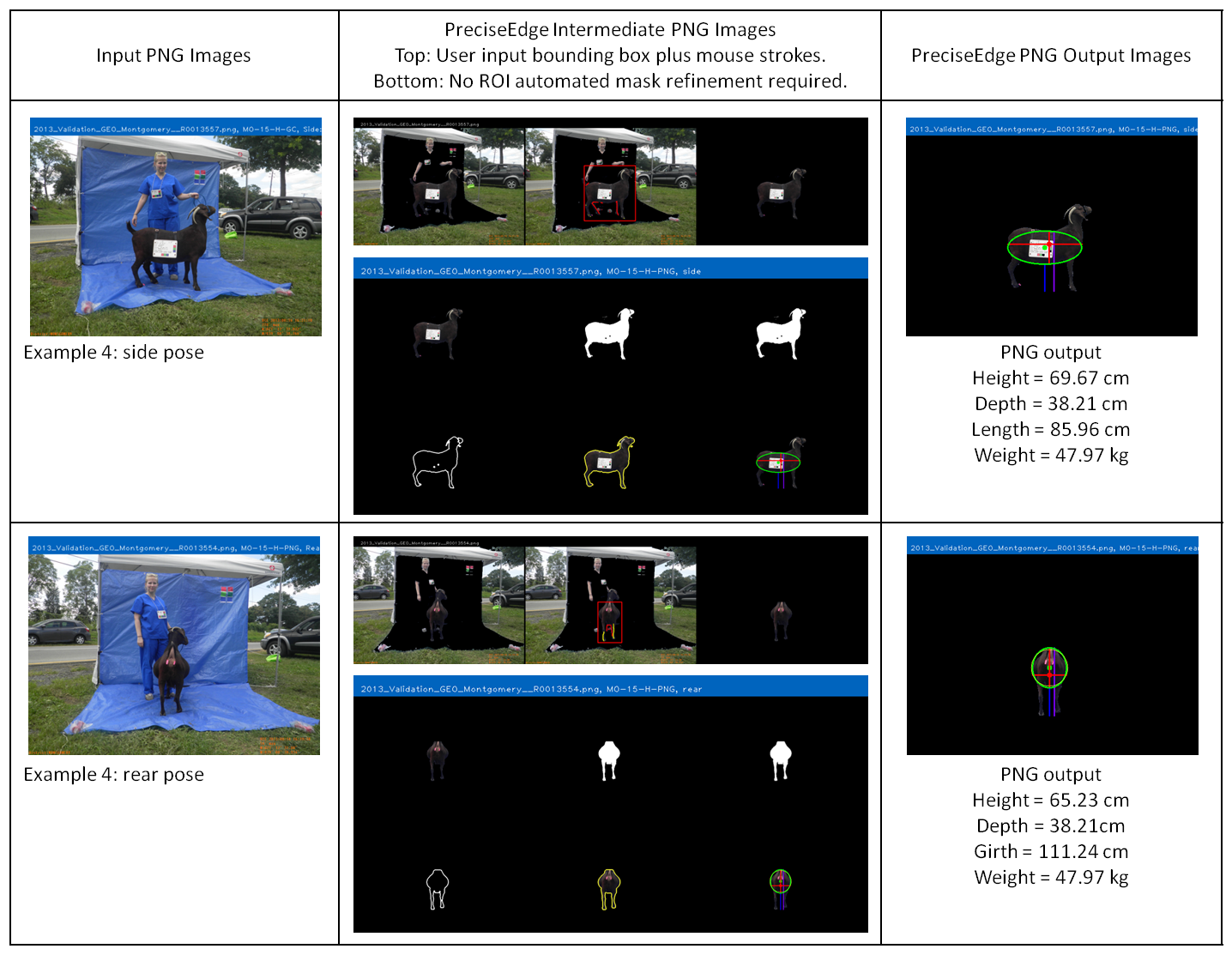

Supplement: S2 Table — (DOCX) [file pone.0275821.s002.docx]
